# Role of ethnicity and environment on lifestyle and cardiometabolic profile in the Native American Mapuche population: Protocol for a systematic review and meta-analysis

**DOI:** 10.1097/MD.0000000000013354

**Published:** 2018-11-30

**Authors:** José C. Fernández-Cao, Carlos Doepking

**Affiliations:** Department of Nutrition and Dietetics, Faculty of Health Sciences, University of Atacama, Copiapó, Chile.

**Keywords:** cardiometabolic profile, ethnic groups, lifestyle, Mapuche, meta-analysis, systematic review, urbanization

## Abstract

**Introduction:**

Cardiometabolic risk factors contribute to increase the incidence of some of the most widespread noncommunicable diseases today. The propensity to present an elevated cardiometabolic risk appears to especially affect non-Caucasians ethnic groups, such as the Native American Mapuche population. In addition, urbanization process has also a great impact on lifestyle and cardiometabolic profile. Therefore, a systematic review and meta-analysis will be conducted to summarize the relevant evidence regarding lifestyle and cardiometabolic profile in Mapuche population, and to assess the role of the ethnicity and the urbanization process on the lifestyle and cardiometabolic profile in Mapuche people with respect to European descendants.

**Methods:**

A comprehensive search until 2019 will be performed using PubMed/MEDLINE, SciELO, Web of Science and Scopus. Observational studies, carried out in general population of Mapuche ethnicity living in rural and urban areas, with data of lifestyle and/or cardiometabolic profile, will be selected. A qualitative summary, as well as meta-analyzes when possible, will be done to present the information. Random effects meta-analyzes will be performed using the generic inverse variance method. Pooled effect size will be expressed as mean difference with 95% confidence intervals. Heterogeneity will be assessed using the Cochran *Q*-statistic and the *I*^2^ statistic. Stratified analyzes, meta-regressions, and sensitivity analyzes will be performed to evaluate potential sources of heterogeneity. In addition, random effects meta-regressions and stratified analyzes will also be used to examine the impact of covariates on the effect size estimated. Publication bias will be investigated using Egger and Begg test, and risk of bias will be assessed using the Research Triangle Institute Item Bank (RTI-IB). Finally, the quality of evidence will be evaluated through the Grading of Recommendations Assessment, Development and Evaluation (GRADE) guidelines.

**Results:**

The results of meta-analyzes will provide important information about differences in lifestyle and cardiometabolic profile between Mapuche people and European descendants, as well as between Mapuche people living in urban and rural areas.

**Conclusion:**

This systematic review and meta-analysis will be helpful to obtain a more reliable understanding of lifestyle and cardiometabolic profile in Mapuche population compared to European descendants, and the impact of the transition from rural to urban areas in this ethnic group.

## Abstract 2

Supplemental Digital Content is available in the text

## 1 Introduction

Noncommunicable diseases represent one of the greatest threats to today's society.^[1]^ They are responsible for 41 million deaths annually, 71% of all deaths worldwide.^[2]^ Cardiometabolic risk factors, such as unhealthy lifestyle (unsuitable diets, alcohol consumption, smoking, or lack of physical activity), obesity, insulin resistance, elevated blood pressure, or dyslipidemia, among others, can lead to develop cardiovascular disease, and diabetes mellitus,^[3]^ which account for almost 20 million deaths each year, one-third of all globally.^[2]^

The propensity to present cardiometabolic risk factors appears to disproportionally affect non-Caucasians ethnic groups. Data compiled from several studies have shown that indigenous from Australia, United States, and New Zealand seem to have more cardiometabolic risk factors than their respective nonindigenous counterparts.^[4]^ Thus, all these indigenous populations presented higher rates of obesity and diabetes mellitus, as well as higher rates of smoking, alcohol abuse, and lower consumption of fruits and vegetables compared to their respective nonindigenous counterparts. Higher blood pressure was also found for Maori and Aboriginal Australians; and higher cholesterol levels for indigenous groups in the United States, compared to nonindigenous people.^[4]^ Likewise, one of the most prominent and renowned cases is Pima Indians, of which was observed a higher insulin resistance compared to Caucasians,^[5]^ and the highest prevalence of type 2 diabetes mellitus ever reported in the world.^[6]^

Another ethnic group that has also been a source of interest regarding their lifestyle, and health condition, is the Mapuche people.^[7–13]^ These natives of South America represent around 10% and 0.5% of the Chilean (1.75 million), and Argentine (0.2 million) population, respectively; and they are the largest indigenous groups in both countries, according to the latest censuses. Observational studies conducted in subjects of this ethnic group showed greater susceptibility to develop obesity,^[11]^ diabetes mellitus,^[7]^ hypertension,^[8]^ and metabolic syndrome^[9,10]^ compared to descendants of Europeans. It is likely that, in common with other indigenous groups, the Mapuche population may have particular factors that predispose them to an increased risk of cardiometabolic diseases. Thus, an interesting study carried out by Celis-Morales et al showed, for the 1st time, higher insulin resistance and levels of insulin and leptin^[7]^ in individuals of Mapuche ethnicity with respect to subjects of European descent. Nevertheless, not all studies have found an increased cardiometabolic risk in this ethnic group. Previous studies had found even significant lower blood levels of glucose^[11]^ and leptin^[13]^ in children and adults of Mapuche ethnicity, respectively. Regarding lipid profile, Casanueva et al also observed significantly lower levels of total cholesterol and triglycerides in rural children of Mapuche ethnicity compared to their respective nonindigenous counterparts.^[14]^ Similarly, lower triglycerides concentration were found in rural adults of Mapuche ethnicity by Celis-Morales et al, in addition to higher levels of high-density lipoprotein (HDL) cholesterol, in comparison with rural adults descendants of Europeans.^[7]^ Residing in rural environment could have a beneficial effect on cardiometabolic risk factors for Mapuche people, as Casanova suggested.^[14]^ More recent studies showed no significant differences in systolic and diastolic blood pressure in individuals older than 15 years,^[7,12]^ but a higher prevalence of hypertension was found in urban schoolchildren of Mapuche ethnicity^[8]^ compared to their respective counterparts of European descent. Differences in anthropometric measures between both groups remains also unclear.^[7,15–18]^ However, an increment in weight, body mass index (BMI), waist circumference,^[9]^ as well as percentage of fat body mass,^[7]^ was observed in subjects of Mapuche ethnicity due to the shifting from rural to urban areas.

There is strong evidence showing that the urbanization process has a great impact in lifestyle.^[19,20]^ It is known that within urban environments traditional diets are abandoned in favor of a more Western diet.^[20]^ Celis-Morales et al found a lower fiber consumption in urban environments,^[7]^ suggesting a worsening in the quality of the diet when adults of Mapuche ethnicity are changed from rural to urban areas. Previously, García et al observed a significantly higher energy intake and fat consumption in urban with respect to rural indigenous children of Mapuche ethnicity.^[21]^ However, these findings were not corroborated by the study carried out by Celis-Morales et al, who found a similar intake in both urban and rural adults of Mapuche ethnicity.^[7]^ In addition, there were no significant differences in the proportion of smokers or physical activity between subjects of Mapuche ethnicity in comparison to descendants of Caucasians.^[7]^ However, the proportion of smokers was higher, and physical activity was lower in both groups when they resided in urban areas, likely due to the adoption of an urbanized lifestyle. Therefore, the transition from rural to urban areas, and consequently to a westernized lifestyle, may have harmful effects on health status, especially in non-Caucasians ethnic groups, such as the Mapuche people.

For all the aforementioned reasons, a comprehensive, quantitative investigation is necessary and timely to synthesize the existing evidence, and to understand the role of ethnicity and environment on the lifestyle and cardiometabolic profile in the Native American Mapuche population. Therefore, we will implement a systematic review and meta-analysis of observational studies based on the following hypothesis: the ethnic Mapuche population could present differences in lifestyle and have a less healthy cardiometabolic profile compared to descendants of Europeans; and Mapuche people living in urban areas could have a less healthy lifestyle and cardiometabolic profile in contrast to those living in rural areas.

### 1.1 Research aims

The main objectives are to summarize the relevant information regarding the lifestyle and cardiometabolic profile in Mapuche population, and to assess the role of ethnicity and environment on the lifestyle and cardiometabolic profile in this ethnic group in comparison to European descendants. The specific objectives of the systematic review and meta-analysis are:

To collect and describe the lifestyle, through diet, physical activity, smoking, and alcohol consumption; as well as cardiometabolic profile, by means of anthropometric measures, blood pressure, lipid profile, glucose metabolism, iron status, biomarkers of inflammation and cytokines, in Mapuche population.

To compare the lifestyle, through diet, physical activity, smoking, and alcohol consumption; as well as cardiometabolic profile, by means of anthropometric measures, blood pressure, lipid profile, glucose metabolism, iron status, biomarkers of inflammation and cytokines, between individuals of Mapuche ethnicity and descendants of Europeans.

To compare the lifestyle, through diet, physical activity, smoking, and alcohol consumption; as well as cardiometabolic profile, by means of anthropometric measures, blood pressure, lipid profile, glucose metabolism, iron status, biomarkers of inflammation and cytokines, between subjects of Mapuche ethnicity living in rural environments and those living in urban environments.

## 2 Methods

The methodology of this review is reported in this protocol following the Preferred Reporting Items for Systematic Reviews and Meta-Analysis Protocols (PRISMA-P) guidelines (Appendix 1;).^[22]^ This protocol is also registered in the International Prospective Register of Systematic Reviews (PROSPERO). The registration number is CRD42017069924, and can be consulted here: (https://www.crd.york.ac.uk/prospero/display_record.php?RecordID=69924). In addition, the implementation of the systematic review and meta-analysis of observational studies will be conducted in compliance with the MOOSE criteria statement.^[23]^ The project is an ongoing research that is planned to be published in mid-2019.

### 2.1 Eligibility criteria

Studies will be selected if they meet the following inclusion criteria according to study design, participant, interest, comparator, outcome, and setting characteristics.

#### 2.1.1 Study designs

Observational design studies, including cohort, case–control, and cross-sectional studies, will be selected. Other type of observational study design, such as case reports, case series or ecologic, will be discarded. Experimental studies, such as clinical trials, lab or animal studies; as well as reviews and meta-analyzes will also be excluded.

#### 2.1.2 Participants

We will include studies examining general children and adult of Mapuche ethnicity, from 2 years onwards, living in rural and/or urban areas, and their respective counterparts of European descent. It will be considered as Mapuche population all groups that are part of Mapuche ethnicity, such as Pehuenche, Picunches, Huilliches, and Lafkenches. Studies carried out in pregnant women will be excluded.

#### 2.1.3 Exposures

Our interest in this review is to assess the effect of ethnicity (Mapuche vs descendants of Europeans) and urbanization (urban vs rural) on the lifestyle and cardiometabolic profile. Thus, the exposures of interest will be: to belong to the Mapuche ethnic group; as well as, to be part of the Mapuche ethnic group residing in urban areas.

#### 2.1.4 Comparators

We are interested in measuring differences in lifestyle and cardiometabolic profile (detailed bellow) due to belong to the Mapuche ethnic group or population of European descent, as well as due to belong to the Mapuche ethnic group living in urban areas or living in rural areas. Therefore, the comparators will be the descendants of Europeans, in the 1st case, and the Mapuche ethnic group living in rural areas, in the 2nd case.

#### 2.1.5 Outcomes

The primary outcomes of the selected studies will be either lifestyle outcomes or cardiometabolic profile outcomes, in addition to the secondary outcomes, as outlined below. Thus, studies should provide data on the mean and standard deviation (SD), or data that allow their estimation, of the following variables: food consumption, nutrients and energy intake, physical activity, smoking habits, alcohol consumption; as well as anthropometric measures (weight, height, BMI, waist circumference, waist-hip ratio, percentage of fat body mass, percentage of lean body mass, and skin folds), blood pressure (systolic and diastolic blood pressure), lipid profile (total cholesterol, HDL, and low-density lipoprotein [LDL] cholesterol, and triglycerides), glucose metabolism (blood glucose and insulin levels, insulin resistance, hemoglobin A1c [HbA1c]), iron status (serum iron, ferritin, hemoglobin, hepcidin, total iron-binding capacity [TIBC], transferrin), biomarkers of inflammation and cytokines (C-reactive protein [CRP], interleukin [IL]-1, IL-2, IL-6, IL-8, IL-12, IL-16, tumor necrosis factor alpha [TNF-α], retinol-binding protein 4 [RBP4], adiponectin, leptin, and resistin), in subjects of Mapuche ethnicity living in rural and urban environments, and their respective counterparts of European descent. The reporting of either of these outcomes will be mandatory for inclusion.

#### 2.1.6 Setting characteristics

There will be no restrictions by type of setting. All geographical locations will be considered.

#### 2.1.7 Language

Publications reported in the English, Spanish, or other Romance languages will be included. We believe that it would be sufficient given the origin of the population of interest. However, studies in other languages will be translated, if possible, to avoid language restrictions.

### 2.2 Information sources

Four electronic bibliographic databases will be used to find relevant studies: Medical Literature Analysis and Retrieval System Online (MEDLINE), bibliographic database of the USA National Library of Medicine, which will be consulted via PubMed interface from 1946 to present; the Scientific Electronic Library Online (SciELO) database, initiative of the Foundation for the Support of Research of the State of São Paulo, Brazil (FAPESP), and the Latin American and Caribbean Center for Information in Health Sciences (BIREME), which will be reviewed from 1940 onwards; Web of Science (WOS) database, which will be consulted since its inception in 1945 to present; and finally, Scopus, which will be reviewed from 1966 onwards. Scopus and WOS are promoted by a major publisher and a multinational information company, respectively. It is interesting to point out that Scopus and WOS complement each other as neither resource is all inclusive.^[24]^ In addition, SciELO is the main database of journals in Spanish, language in which most of the studies conducted on subjects of Mapuche ethnicity have been published.

### 2.3 Search strategy

Literature search strategy will include searching in different electronic bibliographic databases (MEDLINE, SciELO, WOS, and Scopus), followed by further hand search of reference lists of identified reviews and original publications, and checking the authors’ publications of the included studies, to make sure that all relevant material will be reviewed. In addition, corresponding authors of the potentially eligible publications, as well as the selected ones, will be contacted by email to request relevant data, if necessary.

The search strategy for MEDLINE has been developed 1st, including Medical Subject Headings (MeSH) terms, and then adapted for the other 3 databases (Scopus, WOS, and SciELO). Overall, a combination of subject headings, keywords and free-text terms related to lifestyle (diet, physical activity, smoking habits, alcohol consumption), cardiometabolic profile (anthropometric measures, blood pressure, lipid profile, glucose metabolism, iron status, biomarkers of inflammation, and cytokines), and Mapuche Population, have been used. Moreover, animal studies have been excluded from the search. A health expert with systematic review experience (JCFC) developed the search strategy, with the help of another health expert (CD). The draft of the search strategy for each electronic database is included in Appendix 2;.

### 2.4 Study records

#### 2.4.1 Data management

Two investigators (JCFC and CD) will independently perform the data assessment and extraction of the included studies using a developed data extraction form (Appendix 3;). To reduce bias and errors in data extraction, disagreements will be resolved through discussion until consensus is reached. In the case that the selected studies have missing data, the corresponding authors will be contacted for additional data. To contact the authors, a maximum of 3 email attempts will be sent for each selected study. Data from abstracts published in conference proceedings could be included in the review.

#### 2.4.2 Selection process

Two review authors (JCFC and CD) will independently screen the titles and abstracts for eligibility. If there are studies that could not be clearly included, full documents will be reviewed in a 2nd stage. Once any disagreement occurs, it will be resolved through discussion until consensus is reached among the 2 reviewers. Reasons for excluding studies will be recorded to elaborate the subsequent flow chart. Neither of the reviewers will be blinded to the journal name, in which the studies have been published, nor to the study authors, nor to the institutions involved.

#### 2.4.3 Data collection process

For the data extraction, a standard form containing specified outcomes will be used (Appendix 3;). This form includes information about the characteristics of the selected studies, the population assessed, and the outcomes of interest.

To avoid duplicate data, some strategies will be applied. First, the funding source of all the studies that meet the inclusion criteria will be registered. The data of those publications with the same funding source will be contrasted to detect the possible duplication of information. If the funding source does not appear in the publication, then the list of authors of the matching publications will be contrasted, as well as the geographic location of the data collection. In the event that duplication of data is detected, those articles that present more detailed information on the outcomes of interest and that have a larger sample size, will be selected for the review. If these criteria are not sufficient to make a decision, the most recently published article will be selected.

### 2.5 Data items

It will be collected information about characteristics of the included studies (1st author, year of publication, year of data collection, research group, funding sources, study design, sampling method, matched design, outcome measurement methods, sample size for each group and total, and inclusion criteria for both indigenous and nonindigenous participants), information about the populations studied (age, sex, ethnicity, country, and location, area of residence, socioeconomic status of participants), and outcomes (food consumption, nutrients and energy intake, physical activity, smoking habits, alcohol consumption, weight, height, BMI, waist circumference, hip, waist-hip ratio, percentage of fat body mass, percentage of lean body mass, skin folds, systolic and diastolic blood pressure, total cholesterol, HDL, and LDL cholesterol, triglycerides, blood glucose and insulin levels, insulin resistance, HbA1c, serum iron, ferritin, hemoglobin, hepcidin, TIBC, transferrin, CRP, IL-1, IL-2, IL-6, IL-8, IL-12, IL-16, TNF-α, RBP4, adiponectin, leptin, and resistin) in subjects of Mapuche ethnicity living in rural and urban environments, and their respective counterparts of European descent. Any adjustment for these outcomes will be documented and considered when conducting the meta-analyzes. Extraction form (Appendix 3;) includes all these variables and its definitions, with particular details about the planned outcomes. If data presented in studies are unclear, missing or presented in a form that is either unextractable or difficult to reliably extract, the authors of these studies will be contacted for clarification. Missing data, that finally cannot be obtained, will be noted in the report.

### 2.6 Outcomes and prioritization

The outcomes of interest include descriptive data of the lifestyle (diet, physical activity, smoking habits, and alcohol consumption) and cardiometabolic profile (anthropometric measures, blood pressure, lipid profile, glucose metabolism, iron status, biomarkers of inflammation, and cytokines). Specifically, the primary outcomes of the review will be those which report about lifestyle and the classic components of cardiometabolic profile, such as food consumption, nutrients and energy intake, physical activity, smoking habits, alcohol consumption, weight, BMI, waist circumference, waist-hip ratio, percentage of fat body mass, systolic and diastolic blood pressure, total cholesterol, HDL and LDL cholesterol, triglycerides, blood glucose and insulin levels, insulin resistance, HbA1c, CRP, IL-1, IL-2, IL-6, IL-8, IL-12, and IL-16. The secondary outcomes will include other anthropometric measures (height, hip, percentage of lean body mass, skin folds); biomarkers of iron status (serum iron, ferritin, hemoglobin, hepcidin, TIBC, transferrin), since iron overload has been linked to cardiometabolic disorders, such as type 2 diabetes mellitus,^[25]^ gestational diabetes,^[26]^ metabolic syndrome^[27]^ or cardiovascular disease^[28]^; and certain cytokines (TNF-α, RBP4, adiponectin, leptin, and resistin), which have been related to inflammation and insulin resistance. All outcomes will be presented, preferably, as mean and SD, and their values will be expressed and converted, if necessary, into the International System of Units.

The measure of effect of ethnicity (Mapuche vs descendants of Europeans) and urbanization (urban vs rural) on the lifestyle and cardiometabolic profile will be determined by the mean difference (MD) of the outcomes, between Mapuche ethnic group living in rural areas and those living in urban areas, and between individuals of Mapuche ethnicity and subjects of European descent.

### 2.7 Risk of bias individual studies

Risk of bias in individual studies will be assessed using the Research Triangle Institute Item Bank (RTI-IB) tool for observational studies, including cohort, case–control studies, and cross-sectional studies.^[29,30]^ The RTI-IB, developed from the Agency for Healthcare Research and Quality, consists of 16 items intended to aid in identifying risk of bias, confounding, and precision in individual studies. Taking into account the recommendations of the authors and the study design of the included studies, 9 items will be used for case–control, 12 for cohort, and ten for cross-sectional studies. Each study will be classified as high, unclear, or low risk of bias based on the number of critical appraisal items that are met.

It has been shown that the RTI-IB is more useful to undertake a thorough assessment of the quality of the observational studies than the Newcastle–Ottawa scale (NOS),^[31,32]^ although the latter is still the most used tool currently. In addition, the observed agreement between raters and the inter-rater reliability seem to be higher than the NOS. For all these reasons, it has been decided to use this tool to analyze the risk of bias in the studies included in our review, despite the fact that RTI requires more time and iterative adaptations^[31]^ compared to other tools, such as NOS. Thus, 2 independent reviewers (JCFC, CD) will conduct the quality appraisal, with discrepancies being resolved by discussion.

### 2.8 Data

#### 2.8.1 Data synthesis

Data on the lifestyle and cardiometabolic profile will be presented in tabular and narrative forms to provide an overview of these outcomes, especially for Mapuche population. To determine the impact of ethnicity (Mapuche vs descendants of Europeans) and environment (urban vs rural) on each outcome of lifestyle and cardiometabolic profile, different meta-analyzes will be performed using the generic inverse variance method for combining continuous data. Pooled effect size will be expressed as MD with 95% confidence intervals (CI). Exceptionally, in the case of variables with different measurement scales, we will use the standardized MD with 95% CI. To define the effect size, random-effects models will be used, since it gives more conservative results than fixed-effects models.^[33]^ Random-effects models assumes that differences in effect size could be attributed not only to random error, but also to variation in true exposure effects, that is, heterogeneity.^[34]^ Thus, aspects of a study such as year of publication or data collection, funding sources, study design, sampling method, matched design, bias risk score, outcome measurement methods, sample size and inclusion criteria for both indigenous and nonindigenous participants, clinical and socioeconomic status of participants, geographical location, rural or urban residence, age group, gender, among others, are also expected to vary across each included study, increasing the risk of heterogeneity. In addition, in the case that a small heterogeneity is detected, the 2 approaches would give similar results.^[33]^ Finally, random-effects models take into account not only the between-study variability (heterogeneity), but also the within study variability, expressed by the CI of each study's effect size.^[34]^

Meta-analyzes for each outcome of interest will be ruled out based on: a high heterogeneity (I^2^ > 75%) in the overall pooled estimates and in all the stratified results, or insufficient data available (<2 results). To prevent this last, relevant data presented in forms other than the mean and SD, such as median and the interquartile range, will be estimated using methods proposed by Wan et al, valid for both normal and skewed data.^[35]^ When there are relevant missing data for meta-analyzes, such as means or SDs, it will attempt to get them by contacting the authors of the studies. If missing data cannot be obtained, it will try to reconstruct from other sources, such as *P*-values or *t* statistics.^[36]^ We discard to use imputation techniques, since they involve making assumptions about unknown statistics.^[37]^ In the case that it is not possible to perform a meta-analysis, a qualitative summary will be done to present the information.

Heterogeneity will be tested using the Cochran *Q*-statistic and quantified by the *I*^2^ statistic, which represents the percentage of variation attributable to between-study heterogeneity.^[38]^*I*^2^ values of 25%, 50%, and 75% will be considered as low, medium, and high heterogeneity, respectively.^[39,40]^ Potential sources of heterogeneity will be explored through stratified analyzes, and univariate and multivariate meta-regression models, using covariates, such as the year of publication or data collection of the study, funding sources, study design, sampling method, matched design, bias risk score, outcome measurement methods, sample size and inclusion criteria for both indigenous and nonindigenous participants, clinical and socioeconomic status of participants, geographical location, rural or urban residence, age group, gender, among others. In addition, to examine how much of the heterogeneity is accounted for by these covariates, the adjusted *R*^2^ will be calculated by comparing the baseline value of the heterogeneity variance obtained from the empty regression model, with the heterogeneity variance from the meta-regression, after the covariate is added. Finally, sensitivity analysis will also be performed to assess possible causes of heterogeneity.

Otherwise, depending on the amount of information retrieved, stratified analyzes, and random effects meta-regressions will also be used to examine whether any covariate mentioned above has impact on the effect size estimated. Relevant covariates expressed as a range will be assigned the midpoint of the range to estimate a mean value, to assess their impact through meta-regression models. Bubble plots will be created to show a relevant influence of a single continuous covariate on the effect size. To assess the power of each study on the overall pooled estimates, sensitivity analysis will be conducted using the Leave-One-Out method.^[41]^ This consists of repeating the analysis and removing 1 study each time.

#### 2.8.2 Meta-bias

Publication bias will be quantitatively investigated using Egger^[42]^ and Begg^[43]^ tests, and visualized through funnel plots. Additionally, the trim-and-fill method will be applied to identify and correct the impact of potential publication bias.^[44,45]^ All analyzes will be conducted using STATA statistical software (Version 15.0; STATA Corp, College Station, TX). The significance level will be set at *P* < .05 (*P* < .1 for heterogeneity), and the statistical test will be 2-tailed.

#### 2.8.3 Confidence in cumulative evidence

The quality of evidence for all outcomes will be examined using the Grading of Recommendations Assessment, Development, and Evaluation (GRADE) guidelines. The quality of evidence will be assessed across the domains of risk of bias, applying the RTI-IB tool for observational studies^[29,30]^; consistency, measured through heterogeneity^[46]^; directness, evaluating the relevance of the sample, the outcomes measured and the exposure in the included studies^[47]^; precision, examining the 95% CI, as it provides the optimal primary approach for decisions regarding imprecision^[48]^; and publication bias, using Egger^[42]^ and Begg^[43]^ tests. Definitions for grading the quality of the evidence will be high quality (further research is very unlikely to change our confidence in the estimate of effect), moderate quality (further research is likely to have an important impact on our confidence in the estimate of effect and may change the estimate), low quality (further research is very likely to have an important impact on our confidence in the estimate of effect and is likely to change the estimate), or very low quality (any estimate of effect is very uncertain).^[49]^

#### 2.8.4 Ethics and dissemination

This systematic review and meta-analysis has no requirement of ethical approval and informed consent, because it includes no confidential personal data or interventions with the patients. The results of this systematic review will be disseminated only in a peer-reviewed publication or conference.

## 3 Discussion

To the best of our knowledge, no reviews have been carried out regarding the lifestyle and cardiometabolic profile in the Native American Mapuche population. This systematic review and meta-analysis will be helpful to synthesize the existing evidence and get a more reliable understanding of diet, physical activity, smoking habits, alcohol consumption, as well as anthropometric measures, blood pressure, lipid profile, glucose metabolism, iron status, inflammation and cytokines profile, in this ethnic group. In addition, a comprehensive research will be performed to ascertain whether subjects of Mapuche ethnicity, actually, have a less healthy cardiometabolic profile compared to descendants of Europeans, and therefore a greater propensity to develop noncommunicable diseases, such as cardiovascular disease, diabetes mellitus or metabolic syndrome. Data compiled from other studies have suggested that indigenous populations may present a higher cardiometabolic risk than individuals of Caucasian origin.^[4–6]^ This systematic review and meta-analysis will enable to contrast whether this also happens in subjects of Mapuche ethnicity, as other non-Caucasian ethnic groups.

Furthermore, strong evidence exists about the harmful effect of the transition from rural to urban areas on lifestyle^[19,20]^ and health condition.^[50]^ Thus, urbanization has been linked with higher incidence of obesity, diabetes mellitus, or hypertension.^[50]^ Among others, the causes may be the westernization of lifestyle, characterized by the abandonment of traditional diets and the decrease in physical activity.^[20]^ This study will also serve to determine the impact of the urbanization process on the lifestyle and cardiometabolic profile in Mapuche people, who traditionally lived in rural areas, far from the cities.

In conclusion, the present systematic review and meta-analysis will report important information about lifestyle and cardiometabolic profile in Mapuche population, also it will define potential differences regarding European descendants. In addition, it will assess the impact of urbanization process in this ethnic group. Furthermore, the study will aid to identify where future research would be required with respect to lifestyle or cardiometabolic profile in Mapuche people. For instance, this research may inform about possible differences in cardiometabolic profile between Mapuche population and European descendants, and as a result, further researches would be necessary to confirm this finding and explore the cause.

## Acknowledgments

The authors thank the project ATA1756 of the Ministry of Education of Chile. They also thank the university's teacher of English, Ximena Muñoz, for her support in this manuscript.

## Author contributions

JCFC is the guarantor. JCFC conceived, designed and drafted the protocol and provided statistical expertise. JCFC and CD developed the search strategy. CD contributed to the conception and the development of the protocol. All authors read, provided feedback and approved the final protocol.

**Conceptualization:** José Cándido Fernández-Cao, Carlos Doepking.

**Formal analysis:** José Cándido Fernández-Cao.

**Funding acquisition:** José Cándido Fernández-Cao.

**Investigation:** José Cándido Fernández-Cao, Carlos Doepking.

**Methodology:** José Cándido Fernández-Cao.

**Project administration:** José Cándido Fernández-Cao.

**Writing – original draft:** José Cándido Fernández-Cao.

**Writing – review & editing:** José Cándido Fernández-Cao, Carlos Doepking.

José Cándido Fernández-Cao orcid: 0000-0002-1697-3443.

Carlos Doepking Mella orcid: 0000-0001-6281-2686.

## Supplementary Material

Supplemental Digital Content
