# Role of ethnicity and environment on lifestyle and cardiometabolic profile in the Native American Mapuche population: Protocol for a systematic review and meta-analysis: Erratum

**DOI:** 10.1097/MD.0000000000014357

**Published:** 2019-01-25

## Abstract

Supplemental Digital Content is available in the text

In the article, “Role of ethnicity and environment on lifestyle and cardiometabolic profile in the Native American Mapuche population: Protocol for a systematic review and meta-analysis”,^[1]^ which appeared in Volume 97, Issue 48 of *Medicine*, the Extraction Form was not included with the appendices and can be found at.

## Supplementary Material

Supplemental Digital Content
