# Supplementary material for: Role of ethnicity and environment on lifestyle and cardiometabolic profile in the Native American Mapuche population: Protocol for a systematic review and meta-analysis
Source: Medicine (Baltimore). 2018 Nov 30;97(48):e13354. doi: 10.1097/MD.0000000000013354 (PMC6283115; doi:10.1097/MD.0000000000013354)
Supplement: Supplemental Digital Content [file medi-97-e13354-s001.pdf]

APPENDIX 1: PRISMA-P Checklist

APPENDIX 2: Search strategy

APPENDIX 3: Extraction Form (please, find the Excel document in the first submission)

APPENDIX 4: Financial Conflicts of Interest Forms

Section and topic	Item	Checklist item	Reported on page
		**ADMINISTRATIVE INFORMATION**	
Title:			
Identification	1a	Identify the report as a protocol of a systematic review.	(Page 1, title page)
Update	1b	If the protocol is for an update of a previous systematic review, identify as such.	(N/A)
Registration	2	If registered, provide the name of the registry (such as PROSPERO) and registration number.	(Page 4)
Authors:			
Contact	3a	Provide name, institutional affiliation, e-mail address of all protocol authors; provide physical mailing address of corresponding author.	(Page 1, title page)
Contributions	3b	Describe contributions of protocol authors and identify the guarantor of the review.	(Page 11)
Amendments	4	If the protocol represents an amendment of a previously completed or published protocol, identify as such and list changes; otherwise, state plan for documenting important protocol amendments.	(N/A)
Support:			
Sources	5a	Indicate sources of financial or other support for the review.	(Page 11)
Sponsor	5b	Provide name for the review funder and/or sponsor.	(Page 11)
Role of sponsor or funder	5c	Describe roles of funder(s), sponsor(s), and/or institution(s), if any, in developing the protocol.	(Page 11)
		**INTRODUCTION**	
Rationale	6	Describe the rationale for the review in the context of what is already known.	(Pages 3 and 4)
Objectives	7	Provide an explicit statement of the question(s) the review will address with reference to participants, interventions, comparators, and outcomes (PICO).	(Page 4)
		**METHODS**	
Eligibility criteria	8	Specify the study characteristics (such as PICO, study design, setting, time frame) and report characteristics (such as years considered, language, publication status) to be used as criteria for eligibility for the review.	(Page 5)
Information sources	9	Describe all intended information sources (such as electronic databases, contact with study authors, trial registers or other grey literature sources) with planned dates of coverage.	(Page 6)
Search strategy	10	Present draft of search strategy to be used for at least one electronic database, including planned limits, such that it could be repeated.	(Page 6)
Study records:			
Data management	11a	Describe the mechanism(s) that will be used to manage records and data throughout the review.	(Page 6)
Selection process	11b	State the process that will be used for selecting studies (such as two independent reviewers) through each phase of the review (that is, screening, eligibility and inclusion in meta-analysis).	(Page 7)
Data collection process	11c	Describe planned method of extracting data from reports (such as piloting forms, done independently, in duplicate), any processes for obtaining and confirming data from investigators.	(Page 7)

Data items	12	List and define all variables for which data will be sought (such as PICO items, funding sources), any pre-planned data assumptions and simplifications.	(Pages 7 and 8)
Outcomes and prioritization	13	List and define all outcomes for which data will be sought, including prioritization of main and additional outcomes, with rationale.	(Pages 7 and 8)
Risk of bias in individual studies	14	Describe anticipated methods for assessing risk of bias of individual studies, including whether this will be done at the outcome or study level, or both; state how this information will be used in data synthesis.	(Pages 8)
Data synthesis	15a	Describe criteria under which study data will be quantitatively synthesised.	(Pages 8 and 9)
	15b	If data are appropriate for quantitative synthesis, describe planned summary measures, methods of handling data and methods of combining data from studies, including any planned exploration of consistency (such as I^2, Kendall's τ).	(Page 9)
	15c	Describe any proposed additional analyses (such as sensitivity or subgroup analyses, meta-regression).	(Page 9)
	15d	If quantitative synthesis is not appropriate, describe the type of summary planned	(Page 8)
Meta-bias(es)	16	Specify any planned assessment of meta-bias(es) (such as publication bias across studies, selective reporting within studies).	(Page 9)
Confidence in cumulative evidence	17	Describe how the strength of the body of evidence will be assessed (such as GRADE).	(Page 10)

Search strategy

Set	Purpose	Search
1	Anthropometry	weight OR body size OR height OR BMI OR body mass index OR overweight OR obesity OR waist circumference OR waist-hip ratio OR fat mass OR body fat OR lean tissue OR lean mass OR skin folds OR skin fold OR skinfold
2	Diet	diet OR feeding OR nutrition OR food intake OR dietary intake OR feeding behavior OR food OR drink OR beverages OR "Diet, Food, and Nutrition"[Mesh]
3	Other Lifestyle Components	physical activity OR physical inactivity OR sedentary lifestyle OR smoking OR alcohol OR alcohol drinking OR alcoholism
4	Blood Pressure	blood pressure OR hypertension OR cardiovascular diseases OR cardiovascular
5	Lipid Profile	lipidic profile OR lipid profile OR triglycerides OR hypertriglyceridemia OR cholesterol OR total cholesterol OR LDL OR HDL OR hypercholesterolemia
6	Glucose Metabolism	glucose OR hemoglobin glycated OR HbA1c OR glucose metabolism disorders OR glycemia OR hyperglycemia OR insulin OR diabetes OR insulin resistance OR HOMA OR homeostatic model assessment OR metabolic syndrome OR syndrome X
7	Iron Status	iron OR ferritin OR hemoglobin OR anemia OR hepcidin OR TIBC OR transferrin
8	Inflammation and Cytokines	inflammation OR C Reactive Protein OR CRP OR Interleukin OR IL1 OR IL-1 OR IL2 OR IL-2 OR IL6 OR IL-6 OR IL8 OR IL-8 OR IL12 OR IL-12 OR IL16 OR IL-16 OR Tumor Necrosis Factor-alpha OR TNF-alpha OR TNFα OR retinol-binding protein 4 OR RBP4 OR adipokines OR adiponectin OR leptin OR resistin
9	Lifestyle and Cardio-metabolic Profile	OR 1-8
10	Mapuche Population	Mapuche OR Pehuenche OR Araucanian OR Chilean native OR Argentine native OR Chilean ethnicity OR Argentine ethnicity OR Chilean aboriginal OR Argentine aboriginal OR Chilean indigenous OR Argentine indigenous OR Chilean Amerindian OR Argentine Amerindian OR Chilean indians OR Argentine indians OR "Indians, South American"[Mesh]
11	Lifestyle, Cardio-metabolic Profile and Mapuche Population	AND 9-10
12	Human Limit	animals
13	Final Result	NOT 11-12

Table 1: Financial Conflicts of Interest Checklist 2010

(Underlined terms are defined in the Glossary)

The Financial Conflicts of Interest Checklist 2010 was designed to be completed by each investigator in the context of a specific clinical research study.

As awareness of financial conflict of interest issues grows, we see the checklist being completed by other study team members, such as study coordinators, research assistants and study nurses.

This checklist contains four sections: administrative information, study information, personal financial information, and authorship information. The investigator is expected to complete the checklist prospectively as the clinical research moves through its various stages. Sections 1, 2 and 3 are first filled out at the study's initiation, updated as required, and completed when the study manuscript is submitted for publication; section 4 is also completed at this time.

SECTION 1: ADMINISTRATIVE INFORMATION

This section is completed at the study's initiation and updated as necessary.

MODULE A: ADMINISTRATIVE PROFILE

ITEM	DESCRIPTOR	RESPONSE
**A.1.0**	**Study** — **Role of ethnicity and environment on lifestyle and cardio-metabolic profile in Mapuche population: A protocol for systematic review and planned meta-analysis.**	
**A.1.1**	Study name	
**A.1.2**	■ Single site or ❑ multi-site	
**A.1.3**	Countries in which the data will be collected	Chile
**A.1.4**	Is this a clinical trial?	❑ Yes ■ No
A.1.4a	If you answered yes to item A.1.4:	
	Is the study registered in a primary clinical trial registry that follows international standards developed by the World Health Organization and endorsed by the International Committee of Medical Journal Editors?	❑ Yes ❑ No ❑ Don't know
	A list of approved registries can be found at http://www.who.int/ictrp/network/primary/en/index.html	
A.1.4b	What is the primary registry name and the registration number?	
A.1.5	Name of the institution from which the study will be coordinated	Atacama University
A.1.6	Is any part of the study to be conducted by a contract research organization?	❑ Yes ■ No
**A.2.0**	**Investigator**	
A.2.1	Name of the overall study official	José C. Fernández Cao
A.2.2	Name of the investigator completing the checklist	José C. Fernández Cao
A.2.3	What is your role in this research study? (check all that apply)	
A.2.3a	Principal investigator for the entire study	■ Yes ❑ No
A.2.3b	Principal investigator for a site or region	❑ Yes ■ No
A.2.3c	Co-investigator for the study	❑ Yes ■ No
A.2.3d	Paid consultant for the study	❑ Yes ■ No
A.2.3e	Member of steering committee	❑ Yes ■ No
A.2.3f	Participant recruiter	❑ Yes ■ No
A.2.3g	Other (please specify)	

Date the checklist section 1 was first completed (day/month/year) 19/10/2018

Date(s) the checklist section 1 was updated (day/month/year)

SECTION 2: STUDY INFORMATION

This section is completed at the study's initiation and updated as necessary.

MODULE B: FUNDER PROFILE

ITEM	DESCRIPTOR	RESPONSE
**B.1.0**	**Is this study funded?**	■ Yes ☐ No ☐ Don't know
**B.1.1**	If you answered yes to item B.1.0, identify the type of funding support: ■ Financial ☐ Equipment ☐ Test kit ☐ Drug ☐ Device ☐ Other (please specify:________________)	The principal investigator (JCFC) is supported by the Project ATA1756 of the Ministry of Education of Chile.
B.1.2	List the funder(s)	
B.1.3	To which categories do/does the funder(s) belong? (check all that apply):	
B.1.3a	Industry (e.g., pharmaceutical company, test or medical device company, biotech company)	☐ Yes ■ No
B.1.3b	Government funding agency (e.g., National Institutes of Health, Canadian Institutes of Health Research, Medical Research Council)	☐ Yes ■ No
B.1.3c	National or regional government body (e.g., National Health Service, Ministry of Health, Department of Defense)	■ Yes ☐ No
B.1.3d	Charitable foundation (e.g., American Heart Association, The Bill & Melinda Gates Foundation, Wellcome Trust)	☐ Yes ■ No
B.1.3e	Other(s) (please specify:________________)	☐ Yes ■ No

MODULE C: CONTRACT PROFILE

ITEM	DESCRIPTOR	RESPONSE
**C.1.0**	**Is there a contract with the funder(s)?**	☐ Yes ■ No ☐ Don't know
	(If you answered no or don't know, skip to module D)	
	If you answered yes to item C.1.0, does your contract:	
C.1.1	include someone signing on behalf of your institution?	☐ Yes ☐ No
C.1.2	require you to obtain additional funds for this research study from other sources?	☐ Yes ☐ No
C.1.3	contain a clause that prohibits you from disclosing certain aspects about the study without the permission of the funder?	☐ Yes ☐ No
C.1.4	specify the maximum allowable time for pre-publication review by the funder?	☐ Yes ☐ No
C.1.4a	If you answered yes to item C.1.4, what is that time?	______days

MODULE D: STUDY TEAM AND FUNDER RELATIONSHIP PROFILE

ITEM	DESCRIPTOR	RESPONSE			
**D.1.0**	**Who bears final responsibility for and/or has ultimate authority over the following areas of the study?**				
D.1.1	Conceptualizing and designing the study *†	■ Study team	❑ Funder	❑ Shared§	❑ Don't know
D.1.2	Approving the final design†	■ Study team	❑ Funder	❑ Shared§	❑ Don't know
D.1.3	Approving the final data analysis plan	■ Study team	❑ Funder	❑ Shared§	❑ Don't know
D.1.4	Recruiting participants	■ Study team	❑ Funder	❑ Shared§	❑ Don't know
D.1.5	Collecting or assembling data*†	■ Study team	❑ Funder	❑ Shared§	❑ Don't know
D.1.6	Analyzing the data*†	■ Study team	❑ Funder	❑ Shared§	❑ Don't know
D.1.7	Interpreting the data*†	■ Study team	❑ Funder	❑ Shared§	❑ Don't know
D.1.8	Supervising or coordinating the study	■ Study team	❑ Funder	❑ Shared§	❑ Don't know
D.1.9	Deciding on the dissemination plan related to study results	■ Study team	❑ Funder	❑ Shared§	❑ Don't know
D.1.10	If the study is published, who bears final responsibility for and/or has ultimate authority over the following areas of the manuscript development?				
D.1.10a	Drafting all or parts of the manuscript(s)*†	■ Study team	❑ Funder	❑ Shared§	❑ Don't know
D.1.10b	Revising the manuscript(s) for important intellectual content*†	■ Study team	❑ Funder	❑ Shared§	❑ Don't know
D.1.10c	Giving final approval of the version to be published*†	■ Study team	❑ Funder	❑ Shared§	❑ Don't know
D.1.10d	Deciding where the manuscript(s) will be submitted for publication†	■ Study team	❑ Funder	❑ Shared§	❑ Don't know
D.1.10e	Deciding the timing of the manuscript(s) submission for publication†	■ Study team	❑ Funder	❑ Shared§	❑ Don't know
D.1.10f	Deciding authorship	■ Study team	❑ Funder	❑ Shared§	❑ Don't know
D.1.10g	Deciding authorship order‡	■ Study team	❑ Funder	❑ Shared§	❑ Don't know
D.1.10h	Acting as the study guarantor‡	■ Study team	❑ Funder	❑ Shared§	❑ Don't know
D.1.10i	Providing administrative, technical or logistic support	■ Study team	❑ Funder	❑ Shared§	❑ Don't know

* Based on International Committee of Medical Journal Editors (ICMJE), II.A.1. Byline authors, *Uniform requirements for manuscripts submitted to biomedical journals: writing and editing for biomedical publication* (2008).[1] This document describes the ICMJE's three criteria for authorship.

† Based on ICMJE, II.D.2. Potential conflicts of interest related to project support, *Uniform requirements for manuscripts submitted to biomedical journals: writing and editing for biomedical publication* (2008).[1]

‡ Based on World Association of Medical Editors (WAME), Policy statements: authorship.[2]

§ Responsibility and/or authority are shared by the study team and the funder.

Date the checklist section 2 was first completed (day/month/year) 19/10/2018

Date(s) the checklist section 2 was updated (day/month/year) ____________

SECTION 3: PERSONAL FINANCIAL INFORMATION

This section is completed at the study's initiation and updated as necessary.

MODULE E: FINANCIAL PROFILE

ITEM	DESCRIPTOR	RESPONSE
**E.1.0**	**Does this study provide you with salary support?**	☐ Yes ■ No
**E.1.1**	If you answered yes to item E.1.0, what percentage of your annual salary do you estimate will be obtained from the funder(s)?	Not Applicable
**E.2.0**	**Will you personally receive direct or indirect financial benefit for your role in this study?**	■ Yes ☐ No ☐ Don't know
**E.2.1**	If you answered yes to item E.2.0, what is the amount?	$ 712 incentive for publication
**E.3.0**	**Will your department or institution receive or has it received financial benefit (e.g., direct funding, gifts, general use or discretionary funds or any other payment above your institution's standard administrative overhead rate) from the study funder(s)? (check all that apply)**	■ Yes, it does now<br>☐ Yes, it has in the past<br>☐ Yes, it will in the future<br>☐ No<br>☐ Don't know
**E.3.1**	If you answered yes to item E.3.0, please specify the financial benefit:	Don't know
**E.4.0**	**Does this study involve the commercialization of intellectual property (e.g., through patents, copyrights or royalties from such rights)?**	☐ Yes ■ No ☐ Don't know
**E.4.1**	If you answered yes to item E.4.0, who receives the financial benefit from this commercialization?	Not Applicable
**E.4.2**	If you answered yes to item E.4.0, how is the intellectual property commercialized (e.g., through patents, copyrights or royalties from such rights)?	Not Applicable
**E.5.0**	**Do you have any financial interests related to competitor(s) of the funder(s) of your study?**	☐ Yes ■ No
**E.5.1**	If you answered yes to item E.5.0, please specify:	Not Applicable
**E.6.0**	**Do you currently have or expect to have any financial interests related to the study funder(s)?**	☐ Yes ■ No ☐ Don't know
**E.6.1**	If you answered yes to item E.6.0, please specify:	Not Applicable
**E.7.0**	**Do any of your immediate family members (spouse or spouse equivalent, dependent child) currently have or expect to have any financial interests related to the study funder(s)?**	☐ Yes ■ No ☐ Don't know
**E.7.1**	If you answered yes to item E.7.0, please specify:	Not Applicable

Date the checklist section 3 was first completed (day/month/year) 19/10/2018

Date(s) the checklist section 3 was updated (day/month/year) ______

SECTION 4: AUTHORSHIP INFORMATION

This section is completed when a manuscript is being submitted for publication.

MODULE F: AUTHORSHIP PROFILE

ITEM	DESCRIPTOR	RESPONSE
**F.1.0**	**Is there a manuscript submitted for publication?**	❑ Yes ■ No
F.1.1	If you answered yes to item F.1.0, what is the title of the manuscript?	Not Applicable
**F.2.0**	**Are you an author on this manuscript?**	■ Yes ❑ No
F.2.1	To which aspects of the study and the manuscript development did you make a substantial contribution?	
F.2.1a	Obtaining funding‡	■ Yes ❑ No
F.2.1b	Conceptualizing and designing the study*	■ Yes ❑ No
F.2.1c	Providing study materials and/or recruiting participants‡	❑ Yes ■ No
F.2.1d	Collecting or assembling data*	■ Yes ❑ No
F.2.1e	Analyzing and interpreting data*	■ Yes ❑ No
F.2.1f	Providing statistical expertise‡	■ Yes ❑ No
F.2.1g	Supervising or coordinating the study‡	■ Yes ❑ No
F.2.1h	Drafting all or part of the manuscript*	■ Yes ❑ No
F.2.1i	Revising the manuscript for important intellectual content*	■ Yes ❑ No
F.2.1j	Giving final approval of the version to be published*	■ Yes ❑ No
F.2.1k	Providing administrative, technical or logistic support‡	❑ Yes ■ No
F.2.2	Are you the study guarantor?†	■ Yes ❑ No
**F.3.0**	**Are you aware of the involvement of a guest or ghost author?†**	❑ Yes ■ No

* Based on ICMJE, II.A.1. Byline authors, *Uniform requirements for manuscripts submitted to biomedical journals: writing and editing for biomedical publication* (2008).[1] This document describes the ICMJE's three criteria for authorship.

† Based on WAME, Policy statements: authorship.[2]

‡ Derived from the *JAMA* Authorship responsibility, financial disclosure, acknowledgment, and copyright transfer/publishing agreement;[3] some are also mentioned in ICMJE[1] and WAME[2]

Date the checklist section 4 was first completed (day/month/year) 19/10/2018

Date(s) the checklist section 3 was updated (day/month/year) ____________________

Table 1: Financial Conflicts of Interest Checklist 2010

(Underlined terms are defined in the Glossary)

The Financial Conflicts of Interest Checklist 2010 was designed to be completed by each investigator in the context of a specific clinical research study.

As awareness of financial conflict of interest issues grows, we see the checklist being completed by other study team members, such as study coordinators, research assistants and study nurses.

This checklist contains four sections: administrative information, study information, personal financial information, and authorship information. The investigator is expected to complete the checklist prospectively as the clinical research moves through its various stages. Sections 1, 2 and 3 are first filled out at the study's initiation, updated as required, and completed when the study manuscript is submitted for publication; section 4 is also completed at this time.

SECTION 1: ADMINISTRATIVE INFORMATION

This section is completed at the study's initiation and updated as necessary.

MODULE A: ADMINISTRATIVE PROFILE

ITEM	DESCRIPTOR	RESPONSE
**A.1.0**	**Study**	**Role of ethnicity and environment on lifestyle and cardio-metabolic profile in mapuche population: a protocol for systematic review and planned meta-analysis.**
**A.1.1**	Study name	
**A.1.2**	■ Single site or ☐ multi-site	
**A.1.3**	Countries in which the data will be collected	Chile
**A.1.4**	Is this a clinical trial?	☐ Yes ■ No
A.1.4a	If you answered yes to item A.1.4:	
	Is the study registered in a primary clinical trial registry that follows international standards developed by the World Health Organization and endorsed by the International Committee of Medical Journal Editors?	☐ Yes ☐ No ☐ Don't know
	A list of approved registries can be found at http://www.who.int/ictrp/network/primary/en/index.html	
A.1.4b	What is the primary registry name and the registration number?	CRD42017069924
A.1.5	Name of the institution from which the study will be coordinated	Universidad de Atacama
A.1.6	Is any part of the study to be conducted by a contract research organization?	☐ Yes ■ No
**A.2.0**	**Investigator**	
A.2.1	Name of the overall study official	José Fernández Cao
A.2.2	Name of the investigator completing the checklist	Carlos Doepking Mella
A.2.3	What is your role in this research study? (check all that apply)	
A.2.3a	Principal investigator for the entire study	☐ Yes ■ No
A.2.3b	Principal investigator for a site or region	☐ Yes ■ No
A.2.3c	Co-investigator for the study	■ Yes ☐ No
A.2.3d	Paid consultant for the study	☐ Yes ■ No
A.2.3e	Member of steering committee	☐ Yes ■ No
A.2.3f	Participant recruiter	☐ Yes ■ No
A.2.3g	Other (please specify)	

Date the checklist section 1 was first completed (day/month/year) 19/09/2018

Date(s) the checklist section 1 was updated (day/month/year)

SECTION 2: STUDY INFORMATION

This section is completed at the study's initiation and updated as necessary.

MODULE B: FUNDER PROFILE

ITEM	DESCRIPTOR	RESPONSE
**B.1.0**	**Is this study funded?**	☐ Yes ■ No ☐ Don't know
**B.1.1**	If you answered yes to item B.1.0, identify the type of funding support:<br>☐ Financial ☐ Equipment ☐ Test kit ☐ Drug ☐ Device<br>☐ Other (please specify:________________________)	
B.1.2	List the funder(s)	Not Applicable
B.1.3	To which categories do/does the funder(s) belong? (check all that apply):	
B.1.3a	Industry (e.g., pharmaceutical company, test or medical device company, biotech company)	☐ Yes ■ No
B.1.3b	Government funding agency (e.g., National Institutes of Health, Canadian Institutes of Health Research, Medical Research Council)	☐ Yes ■ No
B.1.3c	National or regional government body (e.g., National Health Service, Ministry of Health, Department of Defense)	☐ Yes ■ No
B.1.3d	Charitable foundation (e.g., American Heart Association, The Bill & Melinda Gates Foundation, Wellcome Trust)	☐ Yes ■ No
B.1.3e	Other(s) (please specify:________________________)	☐ Yes ■ No

MODULE C: CONTRACT PROFILE

ITEM	DESCRIPTOR	RESPONSE
**C.1.0**	**Is there a contract with the funder(s)?**<br>(If you answered no or don't know, skip to module D)<br>If you answered yes to item C.1.0, does your contract:	☐ Yes ■ No ☐ Don't know
C.1.1	include someone signing on behalf of your institution?	☐ Yes ☐ No
C.1.2	require you to obtain additional funds for this research study from other sources?	☐ Yes ☐ No
C.1.3	contain a clause that prohibits you from disclosing certain aspects about the study without the permission of the funder?	☐ Yes ☐ No
C.1.4	specify the maximum allowable time for pre-publication review by the funder?	☐ Yes ☐ No
C.1.4a	If you answered yes to item C.1.4, what is that time?	Not Applicable days

MODULE D: STUDY TEAM AND FUNDER RELATIONSHIP PROFILE

ITEM	DESCRIPTOR	RESPONSE			
**D.1.0**	**Who bears final responsibility for and/or has ultimate authority over the following areas of the study?**				
D.1.1	Conceptualizing and designing the study *†	■ Study team	☐ Funder	☐ Shared§	☐ Don't know
D.1.2	Approving the final design†	■ Study team	☐ Funder	☐ Shared§	☐ Don't know
D.1.3	Approving the final data analysis plan	■ Study team	☐ Funder	☐ Shared§	☐ Don't know
D.1.4	Recruiting participants	■ Study team	☐ Funder	☐ Shared§	☐ Don't know
D.1.5	Collecting or assembling data*†	■ Study team	☐ Funder	☐ Shared§	☐ Don't know
D.1.6	Analyzing the data*†	■ Study team	☐ Funder	☐ Shared§	☐ Don't know
D.1.7	Interpreting the data*†	■ Study team	☐ Funder	☐ Shared§	☐ Don't know
D.1.8	Supervising or coordinating the study	■ Study team	☐ Funder	☐ Shared§	☐ Don't know
D.1.9	Deciding on the <u>dissemination plan</u> related to study results	■ Study team	☐ Funder	☐ Shared§	☐ Don't know
D.1.10	If the study is published, who bears final responsibility for and/or has ultimate authority over the following areas of the manuscript development?				
D.1.10a	Drafting all or parts of the manuscript(s)*†	■ Study team	☐ Funder	☐ Shared§	☐ Don't know
D.1.10b	Revising the manuscript(s) for important intellectual content*†	■ Study team	☐ Funder	☐ Shared§	☐ Don't know
D.1.10c	Giving final approval of the version to be published*†	■ Study team	☐ Funder	☐ Shared§	☐ Don't know
D.1.10d	Deciding where the manuscript(s) will be submitted for publication†	■ Study team	☐ Funder	☐ Shared§	☐ Don't know
D.1.10e	Deciding the timing of the manuscript(s) submission for publication†	■ Study team	☐ Funder	☐ Shared§	☐ Don't know
D.1.10f	Deciding <u>authorship</u>	■ Study team	☐ Funder	☐ Shared§	☐ Don't know
D.1.10g	Deciding <u>authorship order</u>‡	■ Study team	☐ Funder	☐ Shared§	☐ Don't know
D.1.10h	Acting as the study <u>guarantor</u>‡	■ Study team	☐ Funder	☐ Shared§	☐ Don't know
D.1.10i	Providing administrative, technical or logistic support	■ Study team	☐ Funder	☐ Shared§	☐ Don't know

* Based on International Committee of Medical Journal Editors (ICMJE), II.A.1. Byline authors, *Uniform requirements for manuscripts submitted to biomedical journals: writing and editing for biomedical publication* (2008).[1] This document describes the ICMJE's three criteria for authorship.

† Based on ICMJE, II.D.2. Potential conflicts of interest related to project support, *Uniform requirements for manuscripts submitted to biomedical journals: writing and editing for biomedical publication* (2008).[1]

‡ Based on World Association of Medical Editors (WAME), Policy statements: authorship.[2]

§ Responsibility and/or authority are shared by the study team and the funder.

Date the checklist section 2 was first completed (day/month/year) 19/09/2018

Date(s) the checklist section 2 was updated (day/month/year) ____________

SECTION 3: PERSONAL FINANCIAL INFORMATION

This section is completed at the study's initiation and updated as necessary.

MODULE E: FINANCIAL PROFILE

ITEM	DESCRIPTOR	RESPONSE
**E.1.0**	**Does this study provide you with salary support?**	☐ Yes ■ No
**E.1.1**	If you answered yes to item E.1.0, what percentage of your annual salary do you estimate will be obtained from the funder(s)?	Not Applicable
**E.2.0**	**Will you personally receive direct or indirect financial benefit for your role in this study?**	■ Yes ☐ No ☐ Don't know
**E.2.1**	If you answered yes to item E.2.0, what is the amount?	$ 712 by publication
**E.3.0**	**Will your department or institution receive or has it received financial benefit (e.g., direct funding, gifts, general use or discretionary funds or any other payment above your institution's standard administrative overhead rate) from the study funder(s)? (check all that apply)**	☐ Yes, it does now<br>☐ Yes, it has in the past<br>☐ Yes, it will in the future<br>■ No<br>☐ Don't know
**E.3.1**	If you answered yes to item E.3.0, please specify the financial benefit:	Not Applicable
**E.4.0**	**Does this study involve the commercialization of intellectual property (e.g., through patents, copyrights or royalties from such rights)?**	☐ Yes ■ No ☐ Don't know
**E.4.1**	If you answered yes to item E.4.0, who receives the financial benefit from this commercialization?	Not Applicable
**E.4.2**	If you answered yes to item E.4.0, how is the intellectual property commercialized (e.g., through patents, copyrights or royalties from such rights)?	Not Applicable
**E.5.0**	**Do you have any financial interests related to competitor(s) of the funder(s) of your study?**	☐ Yes ■ No
**E.5.1**	If you answered yes to item E.5.0, please specify:	Not Applicable
**E.6.0**	**Do you currently have or expect to have any financial interests related to the study funder(s)?**	☐ Yes ■ No ☐ Don't know
**E.6.1**	If you answered yes to item E.6.0, please specify:	Not Applicable
**E.7.0**	**Do any of your immediate family members (spouse or spouse equivalent, dependent child) currently have or expect to have any financial interests related to the study funder(s)?**	☐ Yes ■ No ☐ Don't know
**E.7.1**	If you answered yes to item E.7.0, please specify:	Not Applicable

Date the checklist section 3 was first completed (day/month/year) 19/09/2018

Date(s) the checklist section 3 was updated (day/month/year) ____________

SECTION 4: AUTHORSHIP INFORMATION

This section is completed when a manuscript is being submitted for publication.

MODULE F: AUTHORSHIP PROFILE

ITEM	DESCRIPTOR	RESPONSE
**F.1.0**	**Is there a manuscript submitted for publication?**	☐ Yes ■ No
F.1.1	If you answered yes to item F.1.0, what is the title of the manuscript?	Not Applicable
**F.2.0**	**Are you an author on this manuscript?**	■ Yes ☐ No
F.2.1	To which aspects of the study and the manuscript development did you make a substantial contribution?	
F.2.1a	Obtaining funding‡	☐ Yes ■ No
F.2.1b	Conceptualizing and designing the study*	■ Yes ☐ No
F.2.1c	Providing study materials and/or recruiting participants‡	☐ Yes ■ No
F.2.1d	Collecting or assembling data*	■ Yes ☐ No
F.2.1e	Analyzing and interpreting data*	■ Yes ☐ No
F.2.1f	Providing statistical expertise‡	☐ Yes ■ No
F.2.1g	Supervising or coordinating the study‡	☐ Yes ■ No
F.2.1h	Drafting all or part of the manuscript*	☐ Yes ■ No
F.2.1i	Revising the manuscript for important intellectual content*	■ Yes ☐ No
F.2.1j	Giving final approval of the version to be published*	■ Yes ☐ No
F.2.1k	Providing administrative, technical or logistic support‡	☐ Yes ■ No
F.2.2	Are you the study guarantor?†	☐ Yes ■ No
**F.3.0**	**Are you aware of the involvement of a guest or ghost author?†**	☐ Yes ■ No

* Based on ICMJE, II.A.1. Byline authors, *Uniform requirements for manuscripts submitted to biomedical journals: writing and editing for biomedical publication* (2008).[1] This document describes the ICMJE's three criteria for authorship.

† Based on WAME, Policy statements: authorship.[2]

‡ Derived from the *JAMA* Authorship responsibility, financial disclosure, acknowledgment, and copyright transfer/publishing agreement;[3] some are also mentioned in ICMJE[1] and WAME[2]

Date the checklist section 4 was first completed (day/month/year) 19/09/2018

Date(s) the checklist section 3 was updated (day/month/year) ____________________
